# Multi-talker background and semantic priming effect

**DOI:** 10.3389/fnhum.2014.00878

**Published:** 2014-10-31

**Authors:** Marie Dekerle, Véronique Boulenger, Michel Hoen, Fanny Meunier

**Affiliations:** ^1^Laboratoire sur le Langage, le Cerveau et la Cognition, Centre National de la Recherche Scientifique, UMR 5304Lyon, France; ^2^University of LyonLyon, France; ^3^Laboratoire Dynamique Du Langage, Centre National de la Recherche Scientifique, UMR5596Lyon, France; ^4^Centre National de la Recherche Scientifique, UMR5292, Institut National de la Santé et de la Recherche Médicale, U1028, Lyon Neuroscience Research Center, Brain Dynamics and Cognition TeamLyon, France

**Keywords:** semantic priming, informational masking, cocktail party, cognitive load, effortfulness hypothesis

## Abstract

The reported studies have aimed to investigate whether informational masking in a multi-talker background relies on semantic interference between the background and target using an adapted semantic priming paradigm. In 3 experiments, participants were required to perform a lexical decision task on a target item embedded in backgrounds composed of 1–4 voices. These voices were Semantically Consistent (SC) voices (i.e., pronouncing words sharing semantic features with the target) or Semantically Inconsistent (SI) voices (i.e., pronouncing words semantically unrelated to each other and to the target). In the first experiment, backgrounds consisted of 1 or 2 SC voices. One and 2 SI voices were added in Experiments 2 and 3, respectively. The results showed a semantic priming effect only in the conditions where the number of SC voices was greater than the number of SI voices, suggesting that semantic priming depended on prime intelligibility and strategic processes. However, even if backgrounds were composed of 3 or 4 voices, reducing intelligibility, participants were able to recognize words from these backgrounds, although no semantic priming effect on the targets was observed. Overall this finding suggests that informational masking can occur at a semantic level if intelligibility is sufficient. Based on the Effortfulness Hypothesis, we also suggest that when there is an increased difficulty in extracting target signals (caused by a relatively high number of voices in the background), more cognitive resources were allocated to formal processes (i.e., acoustic and phonological), leading to a decrease in available resources for deeper semantic processing of background words, therefore preventing semantic priming from occurring.

## Introduction

In daily life, speech is rarely perceived in silence, but with interference from wind, music or other people's conversation. Although often used to study psychoacoustic topics (Brungart, 2001; Brungart et al., 2001; McDermott, 2009), speech-in-noise and cocktail party situations (i.e., speech-in-speech, Cherry, 1953) also appear to be interesting paradigms to tackle linguistic processes and competition occurring between backgrounds and targets (Hoen et al., 2007; Boulenger et al., 2010). Our study aimed to investigate the extent to which multi-talker background is processed semantically when listening to speech-in-speech and therefore how the cocktail party situation can be used to study the automaticity of word semantic activation.

The cocktail party situation is described as involving two types of masking effects: energetic and informational masking (Brungart, 2001). Energetic masking relies on the spectro-temporal features of sounds and results from different sounds stimulating the same part of the cochlea at the same time so that one of them cannot be heard (i.e., as two signals increasingly share spectro-temporal characteristics, energetic masking becomes more efficient). In multi-talker background situations, the magnitude of energetic masking is proportional to the number of voices that comprise the background (Simpson and Cooke, 2005). Informational masking, however, usually refers to masking effects that cannot be attributed to energetic masking. Specifically, it is related to the overlap of information carried by the different signals at a higher level (e.g., lexical level and working memory; see Durlach, 2006; Cooke et al., 2008; Mattys et al., 2009; Mattys and Wiget, 2011). Whereas background noise mainly elicits energetic masking, a speech background produces both energetic and informational masking (Brungart et al., 2006). Despite the masking, it is still possible to detect and recognize a word or linguistic token embedded in a babble. Of course, as more voices are present in the babble, participants become less accurate (Freyman et al., 2004). However, it is interesting to note that Simpson and Cooke (2005) showed, using a—6 dB SNR, that intelligibility decreases as a monotonic function of the number of speakers in babbles of up to 8 voices. Specifically, participants' accuracy to detect the target token decreases as the number of voices increases up to 8 voices. Further increasing the number of voices does not lead to a decrease in accuracy. These results suggest that if energetic masking is too high, informational masking decreases with the diminution of the available linguistic cues. For example, with more than 8 talkers, phonetic cues are not or less available and therefore cannot be attributed incorrectly to the target.

The first aim of this paper is to test whether semantic features are involved in informational masking. It has been established that informational masking is not monolithic and occurs at many linguistic levels. Indeed, a multi-talker background will create less interference on a target word, if it is pronounced in a different language (Van Engen and Bradlow, 2007; Brouwer et al., 2012) and different languages will not have the same masking power (Gautreau et al., 2013). By manipulating the number of talkers in the background, Boulenger et al. (2010) revealed lexical competitions between a 2-talker background and target speech using a lexical decision task. Increasing the number of voices in the background, however, led to the disappearance of lexical interference because of increased energetic masking (i.e., words from the background became less intelligible and therefore competed less with target processing). However, using the same paradigm but with an intelligibility task, Hoen et al. (2007) showed that lexical processing of a background could be performed with up to 4 concurrent voices; beyond that threshold, masking was too high and seemed to prevent linguistic processes. Although it has been shown that phonological and lexical information contribute to the informational masking effect, our experiments tested the role of semantic information.

Processing of the background's semantic content has already been highlighted with 2 talkers, pronouncing either semantically correct sentences (i.e., “rice is often served in round bowls”) or incorrect sentences (i.e., “the great car met the milk,” Brouwer et al., 2012). Semantic incoherence in the background impacts the recognition of the target sentence. This result suggests that the background signal with 2 talkers is processed semantically. Our experiments aimed to identify how many talkers are allowed in this semantic processing using words and how semantic information from the backgrounds interferes with the identification of target words.

The ability to semantically process auditory words presented outside of the attentional focus is traditionally studied using dichotic listening. This paradigm allows to study pure informational masking as no energetic masking occurs in dichotic listening. However, discrepant results have been reported (Cherry, 1953; Lewis, 1970; Eich, 1984; Wood et al., 1997; Dupoux et al., 2003). In 1984, Eich showed a semantic effect on the recognition of words presented in the unattended channel. However, this effect resulted, at least partially, from an attentional shift toward the to-be-ignored channel (as suggested by Wood et al., 1997). As the speaker rate was very slow in Eich's experiment (85 words per minute), it allowed participants to listen to the supposedly unattended channel without disturbing the primary task (in the case of Eich's study, a shadowing task). In replicating Eich's study using the same speech rate, Wood et al. (1997) observed the same semantic effect; however, it disappeared if the speaker's rate was increased to 170 words per minute, corresponding to a more ecologically valid rate. The authors concluded that as this faster speech rate demanded more cognitive resources, participants could no longer shift attention to the unattended channel while performing the primary task, suggesting that at least in dichotic listening, informational masking does not involve semantic information. The issue raised by this paradigm is that the spatial separation of auditory signals creates a masking release compared to a binaural condition and therefore facilitates stream segregation that could prevent competition between the to-be-ignored and target speech (Drullman and Bronkhorst, 2000; Hawley et al., 2004).

Concerning semantic activation and according to traditional theoretical models, semantic memory is organized into networks. The recognition of one word leads to its activation in semantic memory, and this activation is supposed to spread automatically to other related concepts (Collins and Quillian, 1969; Collins and Loftus, 1975). This supposition is derived from semantic priming paradigms, shown in auditory and visual modalities, in which the presentation of prime word leads to faster recognition of a semantically related target word (Meyer and Schvaneveldt, 1971; Donnenwerth-Nolan et al., 1981; Radeau, 1983; Schacter and Church, 1992; Radeau et al., 1998; Spruyt et al., 2012). For example, the presentation of the prime “nurse” before the target “doctor” facilitates the recognition of the target word “doctor” compared to a condition in which the prime is unrelated to the target (Meyer and Schvaneveldt, 1971). Adapting this paradigm to the cocktail party situation will allow us to investigate if the semantic content of the background is processed and interferes with the target word, despite decreased intelligibility. Some background words will therefore act as primes.

In the current study, we used the rationale of a priming paradigm by manipulating the association between words pronounced in the background and target words. Additionally, we varied the amount of masking to evaluate how it modulates semantic priming effects. Participants were required to perform a lexical decision task on a target item (i.e., decide whether the target item is a word or a pseudo-word) embedded in backgrounds composed of 1 to 4 voices depending on the experiment. These voices could pronounce words that were semantically related to each other and that were related or unrelated to the target. They acted as primes and were called Semantically Consistent (SC) voices. Additional voices pronounced words that were always unrelated to each other and unrelated to the target, acting as maskers. They were called Semantically Inconsistent (SI) voices.

Across experiments, we manipulated the ratio between SC and SI voices. The aim was to test the preservation of the semantic processing of SC voices despite increased masking (i.e., more SI voices). In Experiment 1, backgrounds were composed of 1 or 2 SC voices. In Experiments 2 and 3, respectively, 1 and 2 SI voices were added to each background to increase masking and therefore, decrease the intelligibility of the SC voices. Consequently, in Experiment 2, backgrounds in one condition consisted of 1 SC voice and 1 SI voice and in a second condition of 2 SC voices and 1 SI voice. In Experiment 3 they comprised 1 SC voice and 2 SI voices in one condition and 2 SC voices and 2 SI voices in the other condition.

Overall increasing the number of voices allowed us to examine if and how semantic priming can be impacted by the increase in the number of talkers in the background. Additionally, the variation in the number of SC voices compared to the number of SI voices allowed us to study the effect of prime saliency on semantic processing and therefore its participation in informational masking. Indeed, across experiments, backgrounds can consist of the same number of voices whereas the number of SC voices compared to the number of SI voices could differ (e.g., 3 voices in the background: either 2SC/1SI in Experiment 2 or 1SC/2SI in Experiment 3).

If semantic processing can occur automatically, semantic priming should be observed at least as long as background words are intelligible and should not be disturbed by increased masking and decreased prime saliency. Indeed, automaticity is defined as a strategy free processing that occurs without using the resources of a limited capacity central processor (Neely, 1977). Therefore, if semantic processing is strategy free, it should occur even if participants are not aware that a given word is presented to them (as is done in visual modality in classical masked priming paradigms, see Forster and Davis, 1984).

## Experiment 1

The aim of this experiment was to first establish set up and test our paradigm and experimental materials. Backgrounds were composed of 1 or 2 SC voices that pronounced words sharing semantic features with each other. In the related condition, target words belonged to the same semantic field as the prime, but they did not in the unrelated condition. We therefore expected to observe a semantic priming effect: participants should more quickly and accurately identify target words in the related compared to the unrelated condition. The second aim of this first experiment was to test if the presence of 2 voices in the background would affect participants' performance as suggested by the psychoacoustic literature (Brungart, 2001; Brungart et al., 2001). We therefore hypothesized that target words would be answered to more slowly and less accurately in the 2SC condition compared to the 1SC condition. Finally, we examined whether the semantic priming effect was modulated by increased energetic and informational masking caused by the augmentation of the number of voices in the background.

### Method

#### Participants

Twenty-seven participants (20 females) volunteered for this experiment. All were right-handed, French native speakers and reported no known hearing or language disorder. Subjects' ages ranged from 18 to 25 years old. All participants gave written informed consent and were not aware of the experiment's purpose. They were compensated for their participation. The protocol that was used in this experiment was approved by the local ethics committee (CPP Sud-Est IV, Lyon; ID RCB: 2008-A00708-47).

#### Stimuli

Forty-eight disyllabic target words (*M*_lexical frequency_ = 21.94 per million, *SD* = 18.75 according to the French database Lexique 3, New et al., 2001) were selected, and each word belonged to a specific semantic field (e.g., *CAROTTE* “carrot”; *MÉTRO* “subway”). Each target word was matched to 10 words belonging to the same semantic field (e.g., *CAROTTE* “carrot” was associated with *légume, chou, céleri, salade, tomate* “vegetable, cabbage, celery, lettuce, tomato”). As participants had to perform a lexical decision task, 48 pseudo-words respecting French phonotactic rules were created (e.g., *PLARO, HUMEL*). Ten words sharing semantic features with each other were arbitrarily associated with each pseudo-word target, resulting in a total of 96 groups of 10 words (See Supplementary Material) (*M*_lexical frequency_ = 21.86, *SD* = 18.20). As each background comprised 1 or 2 SC voices (related or not to the target), each group was divided into two subgroups of 5 words one of the subgroups was spoken by a first speaker (S1), and the other by a second speaker (S2).

**Figure 1:**
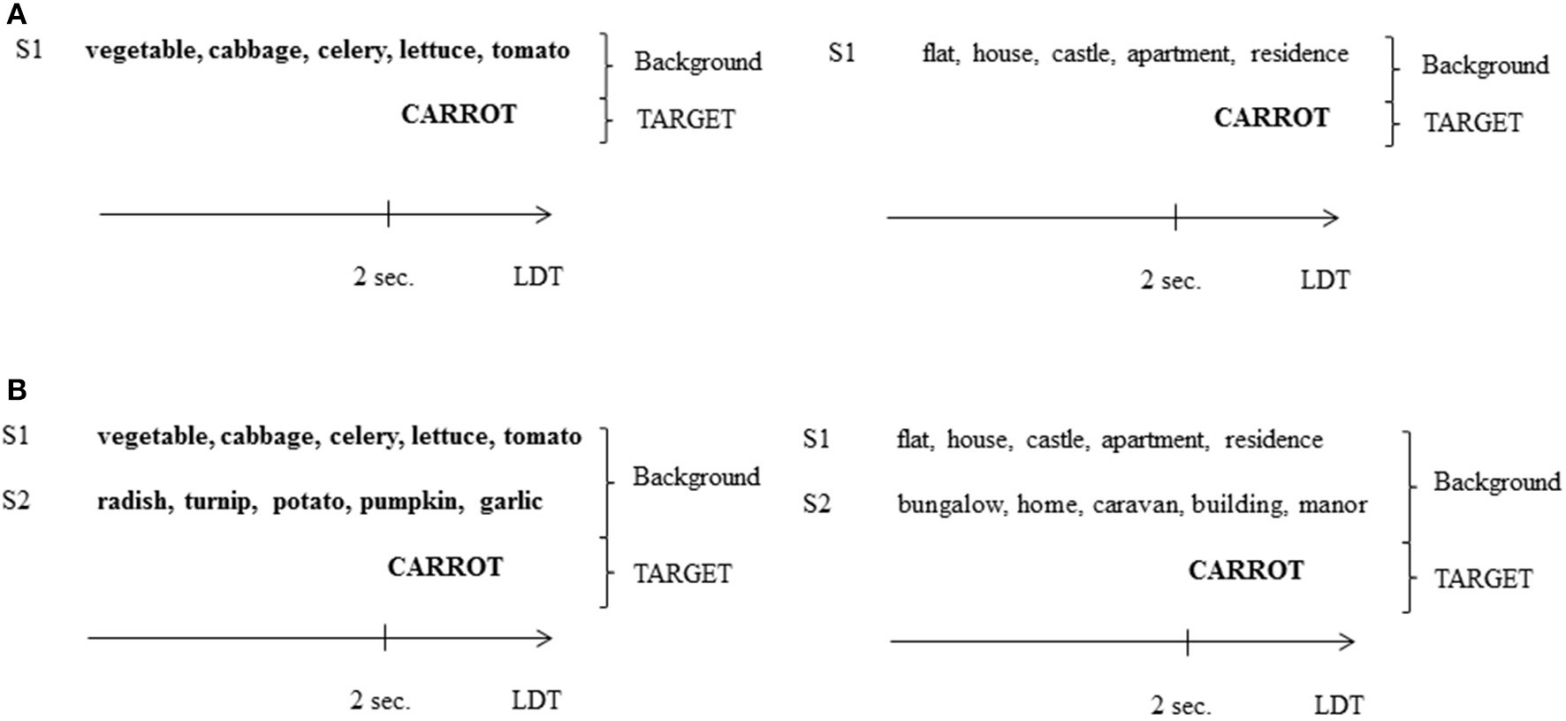
**(A)** Example of two backgrounds in the 1SC condition of Experiment 1, presented with a semantically related target word (left; related condition) or not (right; unrelated condition). S1, speaker 1, LDT, Lexical Decision Task. **(B)** Example of a background in the 2SC condition of Experiment 1, presented with a semantically related target word (left; related condition) or not (right; unrelated condition). S2, speaker 2.

Target words were presented with a semantically related (related condition) or semantically unrelated background (unrelated condition). In the unrelated condition, SC voices pronounced words that were semantically related to each other but not to the target (see Figure 1). Backgrounds comprised 1 SC voice (1SC condition) or 2 SC voices (2SC condition). The 48 target words were divided into 4 groups of 12 words, the mean frequency did not differ significantly between the groups (*F* < 1), nor did the number of phonemes [*M* = 6.97, *SD* = 5.65; *F*_(3, 44)_ = 1.1, *n.s*.] and phonological neighbors [*M* = 4.75, *SD* = 0.81; *F*_(3, 44)_ = 2.2, *n.s*.]. Each group of twelve target words was assigned to a condition (1SC related, 1SC unrelated, 2SC related, 2SC unrelated) depending on the experimental list. The same was true for pseudo-words. Four experimental lists of 96 stimuli (i.e., 48 target words and 48 target pseudo-words) were created so that each target word was presented in each condition, but only once in a list (each participant was presented with one list only).

Targets and SC voices were recorded by 3 different French native female speakers (age: 21–22) in a sound-proof room (22 kHz, mono, 16 bits). Auditory sequences of 5 words from Speakers 1 and 2 (S1 and S2) were segmented into 3 s periods. The periods were then normalized at an intensity of 60 dB-A and mixed together to create backgrounds. All audio files were synchronized at the beginning, so all voices started to speak at the same time. However, as all voices pronounced words of different lengths, they soon became desynchronized, and there was always one speaker talking in the background. Targets recorded by Target Speaker (TS; also normalized at an intensity of 60 dB-A) were inserted 2 s after the start of the backgrounds (so that each participant always had the same exposure to the background before the target speech was presented), with a 0 dB SNR (Signal/Noise Ratio; see Figure 1). Because the backgrounds, which comprised 1 or 2 voices, generated different amounts of energy, the intensity of all stimuli was varied over a ±3 dB range in 1 dB steps to prevent participants from predicting condition depending on individual stimuli intensity.

#### Procedure

Participants sat in front of a computer screen and heard the stimuli binaurally through headphones at a comfortable level (mean level 65 dB-A, ranging from 62 dB-A to 68 dB-A, normalized using an artificial ear). A fixation cross was presented on the screen at the beginning of each trial and remained on the screen during stimulus presentation. Participants were asked to listen to the stimuli to decide as quickly and accurately as possible whether the target was a word or a pseudo-word, by pressing one of two pre-specified keys. After a response was given, a string of hash marks indicated that the trial was over; participants could then press a key to start the next trial. Half of the participants gave the response to “word” with their left hand and to “pseudo-word” with their right hand. As all participants were right-handed, they might answer faster with their right hand than their left hand. To avoid this confounding effect, the other half were given the opposite instruction. A training session composed of twelve trials (different from the experimental stimuli) preceded the test session so that participants could acclimate to the stimuli and the task.

### Results

Two Two-Way repeated measures analyses of variance (ANOVAs) by participants (*F*_1_) and by items (*F*_2_) were conducted, with Response Times (RTs, in ms) and Error Rates (ERs) for target word identification as dependent variables. We included Number of Voices in the background (1 Voice, 1SC or 2 Voices, 2SC) and Semantic Link between prime and target (related or unrelated) as within-subjects factors. Three participants were excluded from analyses because of very high ERs (more than 40%). Four target words error rates greater than 50% were also excluded from Item analyses (*POIGNET, RIDEAU, RATON*, and *RACINE* “wrist, curtain, baby rat, root”). Trials with RTs below or above 2.5 standard deviations from the individual means (4.5%) and trials in which participants made mistakes (19.5%) were not included in RTs analysis. Means and Standard Deviations (SDs) of RTs and ERs are summarized in Table 1.

**Table 1:** **Means and Standard Deviations (SDs) of Response Times (RTs) and Error Rates (ERs) depending on the number of voices in the background and the semantic link between prime and target in Experiment 1**.

		**1SC**		**2SC**	
		**Related**	**Unrelated**	**Related**	**Unrelated**
RT (ms)	Mean	980	1035	1014	1070
	*SD*	170	142	169	150
ER (%)	Mean	7.08	16.33	19.5	20.77
	*SD*	6.82	12.34	19.04	14.05

*1SC, 1 SC voice condition; 2SC, 2 SC voices condition; related, semantic link between the prime and the target; unrelated, no semantic link between the prime and the target*.

The ANOVA by participants first revealed a significant main effect of the Number of Voices: participants were faster [*F*_1(1, 23)_ = 4.25, *p* = 0.05] and more accurate [*F*_1(1, 23)_ = 9.12, *p* < 0.01] to identify targets in the 1SC condition (*M*_RT_ = 1008 ms, *SD* = 157; *M*_ER_ = 11.7%, *SD* = 10.9) than in the 2SC condition (*M*_RT_ = 1042 ms, *SD* = 161; *M*_ER_ = 20.1%, *SD* = 16.5). The Item analysis, however, did not highlight an effect of the Number of Voices on RT [*F*_2(1, 43)_ = 1.9, *p* = 0.1], although target words were better categorized as words in the 1SC condition [*F*_2(1, 43)_ = 13.43, *p* < 0.001].

The main effect of Semantic Link also appeared to be significant on RTs [*F*_1(1, 23)_ = 14.24, *p* < 0.001; *F*_2(1, 43)_ = 4.63, *p* < 0.05], participants responded faster if targets shared semantic features with the prime (*M* = 997 ms, *SD* = 169) than if they did not (*M* = 1053 ms, *SD* = 145); this resulted in a 56 ms priming effect. This effect was also significant for ERs in the participant analysis [*F*_1(1, 23)_ = 3.93, *p* = 0.05], and there was only a trend in the item analysis [*F*_2(1, 43)_ = 3.07, *p* < 0.10]. Participants tended to be more accurate in the related condition (*M* = 15.4%, *SD* = 15.4) than in the unrelated condition (*M* = 18.55%, *SD* = 13.2). There was no significant interaction between the two factors for RTs (*F*_1_ < 1; *F*_2_ < 1) and ERs [*F*_1(1, 23)_ = 2.34, *n.s*.; *F*_2(1, 43)_ = 1.97, *n.s*.], suggesting that the semantic priming effect was not modulated by the Number of Voices (one or two) in the background.

### Discussion

These results first highlight that participants were slowed by the increase in the number of voices in the background. This effect is certainly attributable to enhanced target masking in the two-voice condition (Brungart, 2001; Brungart et al., 2001). Interestingly, participants' performance was improved by the semantic relationship between the prime and target, and this effect was independent of the number of voices, suggesting that the increase in masking from one to two background voices, was not sufficient to prevent semantic processing. However, in this first experiment, prime was salient in both conditions (1SC voice and no SI voice or 2SC voices and no SI voice). To test whether participants could still take advantage of the semantic relationship between target and prime if the intelligibility of the SC voices was further decreased, we conducted a second experiment in which a SI voice was added to each background.

## Experiment 2

This second experiment aimed to investigate whether the semantic priming effect would resist increased masking. An SI voice was therefore added to each background. This voice pronounced words sharing no semantic features with each other or with the target word, whatever the condition. The purpose was to use the same material and procedure as in Experiment 1 with the addition of mask on the SC voices. In Experiment 2, backgrounds were composed of two voices (1 SC voice + 1 SI voice) or 3 voices (2 SC voices + 1 SI voice). A deleterious effect of the number of voices on participants' performance was predicted, and we expected that the presence of SI voice would not affect the semantic priming effect if this latter effect is automatic.

Another change was made in Experiment 2 regarding target items. In Experiment 1, target items were pronounced by a female speaker, and were consequently, difficult to detect among the other female speakers (S1 and S2). These difficulties might partly explain the low accuracy and long response times to target words inserted in babbles that were only composed of one or two voices. To avoid flux segregation difficulties (Festen and Plomp, 1990; Brungart et al., 2001), target items were therefore pronounced by a male speaker (Target Speaker 2; TS2) in the two following experiments.

### Method

#### Participants

Twenty-four right-handed French native speakers (18 females), aged 18–30 years, participated in this second experiment. They had no known auditory or language disorders. All participants gave their written informed consent and were compensated for their participation. None of the participants had been tested in Experiment 1 and they were not aware of the aim of the study before testing.

#### Stimuli

To add an SI voice to each background used in Experiment 1, 96 groups of 5 words (*M*_lexical frequency_ = 18.15, *SD* = 9.75), not semantically related to each other, were generated. Average lexical frequency did not differ between SC voices (from Experiment 1) and the SI voice as shown by an ANOVA [*F*_(2, 190)_ = 1.16, *n.s*.]. Each group was selected to mask a specific prime (composed of 1 or 2 SC voices), which shared no semantic link (e.g., the prime *légume, chou, céleri, salade, tomate* “vegetable, cabbage, celery, lettuce, tomato” was always presented with the SI voice pronouncing *policier, intéressant, cour, affiche, étagère* “policeman, interesting, yard, poster, shelf”). This SI voice was recorded by another French native female speaker (S3, age = 20) using the same method as in Experiment 1.

Backgrounds composed of 1 SC voice (S1) in Experiment 1 were now composed of 1 SC voice and 1 SI voice (S1 + S3), corresponding to the 1SC/1SI condition, and backgrounds composed of 2 SC voices (S1 + S2) in Experiment 1 were now composed of 2 SC voices and 1 SI voice (S1 + S2 + S3), corresponding to the 2SC/1SI condition. The 4 groups of 12 target words and pseudo-words created for Experiment 1 were used. Target words were presented in each of the 4 conditions: 1SC/1SI related, 1SC/1SI unrelated, 2SC/1SI related and 2SC/1SI unrelated. The corresponding number of pseudo-words was also presented. Four experimental lists were created so that each target word was seen in each condition but only once in a list.

**Figure 2:**
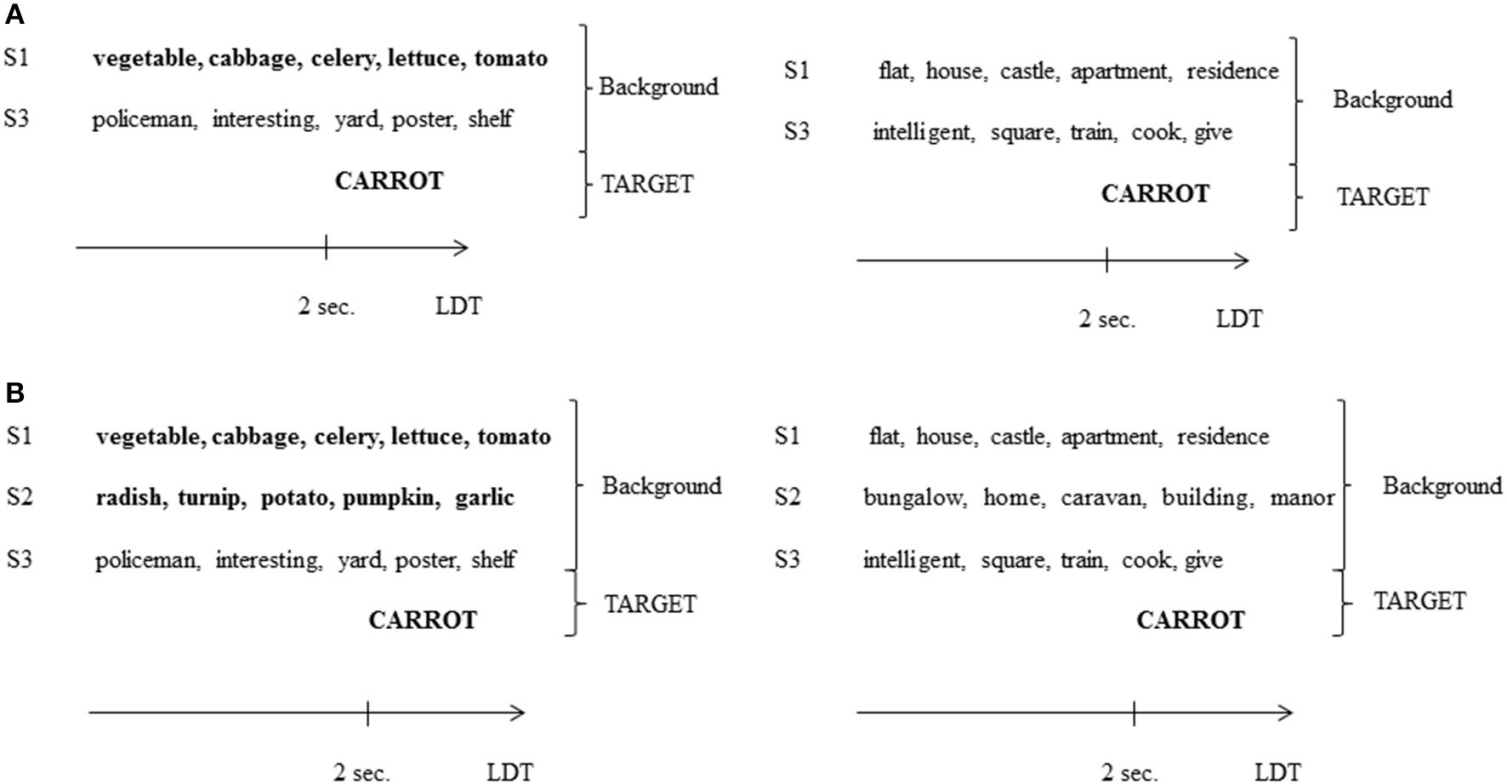
**(A)** Example of a background in the 1SC/1SI condition of Experiment 2, presented with a semantically related target word (left; related condition) or not (right; unrelated condition). S3, speaker 3 (see the legend of Figure 1 for other abbreviations). **(B)** Example of a background in the 2SC/1SI condition of Experiment 2, presented with a semantically related target word (left; related condition) or not (right; unrelated condition).

Recordings of S1 and S2, used in the previous experiment, were mixed with S3 following the previously established experimental lists. Targets were recorded by a French native male speaker (Target Speaker 2; age = 20) and were inserted into backgrounds 2 s after the start of the sequence (see Figure 2).

#### Procedure

The procedure was the same as in Experiment 1.

### Results

Similar analyses as in Experiment 1 were performed by subjects (*F*_1_) and by items (*F*_2_), with RTs (in ms) and ERs for target word identification as the dependent variables. We included Number of Voices in the background (two voices, 1SC/1SI or three voices, 2SC/1SI) and Semantic Link between prime and target (related or unrelated) as within-subjects factors. As in Experiment 1, target words for which less than 50% of participants answered correctly were not analyzed. The target words *BILLET* and *CHIGNON* (“ticket” and “bun”) were therefore not included. Moreover, 15% of data were excluded (13% of errors and 2% of extremes values) from RT analysis. Mean RTs and ERs with SDs are summarized in Table 2.

**Table 2:** **Means and SDs of RTs and ERs depending of the number of voices in the background and the semantic link between prime and target in Experiment 2**.

		**1SC/1SI**		**2SC/1SI**	
		**Related**	**Unrelated**	**Related**	**Unrelated**
RT (ms)	Mean	933	958	945	1000
	*SD*	165	153	162	187
ER (%)	Mean	6.47	10.7	10.7	14.33
	*SD*	7.29	9.14	9.32	8.78

*1SC/1SI, 1 SC voice and 1 SI voice condition; 2SC/1SI, 2 SC voices and 1 SI voice condition*.

The Two-Way repeated measures ANOVAs showed a significant main effect of the Number of Voices in the backgrounds [*F*_1(1, 23)_ = 6.08, *p* < 0.05; *F*_2(1, 45)_ = 4.34, *p* < 0.05]. Participants responded faster to target words if backgrounds comprised 2 voices (*M* = 946 ms, *SD* = 157) compared to 3 voices (*M* = 973 ms, *SD* = 175). This main effect was also significant for ERs [*F*_1(1, 23)_ = 7.18, *p* < 0.05] but only in the participants' analysis [*F*_2(1, 45)_ = 3.72, *p* < 0.10]. Responses were more accurate in the 1SC/1SI condition (*M* = 8.6%, *SD* = 8.2) than in the 2SC/1SI condition (*M* = 12.5%, *SD* = 9.1).

The main effect of Semantic Link was also significant [*F*_1(1, 23)_ = 5.32, *p* < 0.05; *F*_2(1, 45)_ = 5.38, *p* < 0.05], responses were 40 ms faster if the prime and target shared semantic features (*M* = 939 ms, *SD* = 162) compared to if they did not share features (*M* = 979 ms, *SD* = 170). This effect also reached significance for ERs in the participants' analysis [*F*_1(1, 23)_ = 5.33, *p* < 0.05; *F*_2(1, 45)_ = 1.98, *p* = 0.10]. ERs decreased by 3.9% if the prime and target were semantically related (*M* = 8.61%, *SD* = 8.5 in the related condition and *M* = 12.53%, *SD* = 8.8 in the unrelated condition).

There was no significant interaction between the Number of Voices in the backgrounds and the Semantic Link between prime and target for RTs [*F*_1(1, 23)_ = 1.92, *n.s*.; *F*_2_ < 1] and ERs (*F*_1_ < 1; *F*_2_ < 1), indicating that the priming effect was not affected by the increase in the number of voices in the backgrounds.

### Discussion

As in Experiment 1, performances improved if the prime and target were semantically related, although participants were slower in condition 2SC/1SI than 1SC/1SI because of increased masking (Bronkhorst, 2000; Brungart, 2001; Brungart et al., 2001). Interestingly, there was again no indication that increased masking reduced the semantic priming effect. To further test this resistance of the semantic priming effect to prime intelligibility loss, we conducted a third experiment in which a second SI voice was added to each background to further decrease prime saliency. However, the intelligibility of the target item may decrease with the addition of a second SI voice in the background. However, as the target item is pronounced by a male voice and embedded in a female voice background, it is still quite salient compared to background voices (Brungart et al., 2001). Indeed, Brungart and collaborators showed that participants more easily recognize a target sentence if it was embedded in a background composed of voices of a different sex than if all the sentences (background and target) were pronounced by speakers of the same sex. Additionally, in our experiment, target items were presented at the same time (2 s after the beginning of the stimulus), and this regular timing helps participants to detect the target, as they know when to listen to it. Consequently, in our paradigm, the masking effect of the SI voice on the target item was quite low compared to its effect on SC voices.

## Experiment 3

This last experiment was the same as Experiment 2, except that a second SI voice was added to each background (i.e., backgrounds comprised 3 or 4 voices). The same method was used as in Experiment 2. The aim was to further increase the masking of SC voices to test the resistance and automaticity of semantic processing. As the number of voices in the backgrounds increased (i.e., up to 4 voices), we wanted to make sure whether participants could still identify words from the background. Therefore, after the main experiment, we asked participants to perform a recognition test. This post-test was designed to examine whether, in agreement with Hoen et al. (2007), words in the background were still intelligible. It aimed to clarify, in the case of significant semantic priming, if this effect resulted from preserved intelligibility of the prime words by testing lexical access or from automatic processing. In case of preserved intelligibility we cannot prove that the semantic effect results from automatic processing, it may be either automatic or strategic. However, if a priming effect is found without preserved intelligibility, semantic processing is automatic (as shown with masked priming paradigm in visual modality, see Dehaene et al., 1998; Naccache and Dehaene, 2001; Spruyt et al., 2012). Proof of non-intelligibility was consequently needed in the case of a significant semantic priming effect to provide evidence for an automatic process. Otherwise, we would not be able to detect whether automatic components are present in semantic processes. As in our previous experiments, we expected to observe a significant effect of the number of voices in the background; increasing the number of voices has shown to decrease intelligibility for up to 8 voices (Simpson and Cooke, 2005). No interaction between the number of voices and the semantic association between prime and target was expected, at least if the words composing the background were still intelligible.

### Method

#### Participants

Twenty-four participants (19 females) were recruited for this experiment (age: 18–34). All were right-handed French native speakers and reported no hearing or speech disorder. They gave written informed consent and were compensated for their participation. None of them had participated in Experiments 1 or 2, and they were unaware of the experiment's purpose prior to testing.

#### Stimuli

To create an extra SI voice, pronounced by a fourth speaker (S4), 96 groups of 5 words, that were semantically unrelated to each other, were generated (*M*_lexical frequency_ = 20.88, *SD* = 1.22). The word mean frequency did not differ between the different voices (SC and SI voices; *F* < 1). As in Experiment 2, each group of words was matched to a prime (composed of 1 or 2 SC voices) with which it did not share any semantic features and was systematically presented with (e.g., the prime *légume, chou, céleri, salade, tomate* “vegetable, cabbage, celery, lettuce, tomato” was always presented with the masker *étui, liberté, drôle, global, sympathie* “case, freedom, funny, global, sympathy”). Therefore, with the addition of this second SI voice, backgrounds composed of 2 voices (S1 + S3) from Experiment 2 became 3-voice backgrounds (S1 + S3 + S4; 1 SC voice and 2 SI voices; 1SC/2SI condition) and 3-voice backgrounds (S1 + S2 + S3) from Experiment 2 became 4-voice backgrounds (S1 + S2 + S3 + S4; 2 SC voices and 2 SI voices; 2SC/2SI condition). The 4 groups of 12 target words and pseudo-words created in Experiment 1 were used and presented in the following conditions: 1SC/2SI related, 1SC/2SI unrelated, 2SC/2SI related, and 2SC/2SI unrelated. Four experimental lists were created so that each target word was presented in each condition but only once in a list.

**Figure 3:**
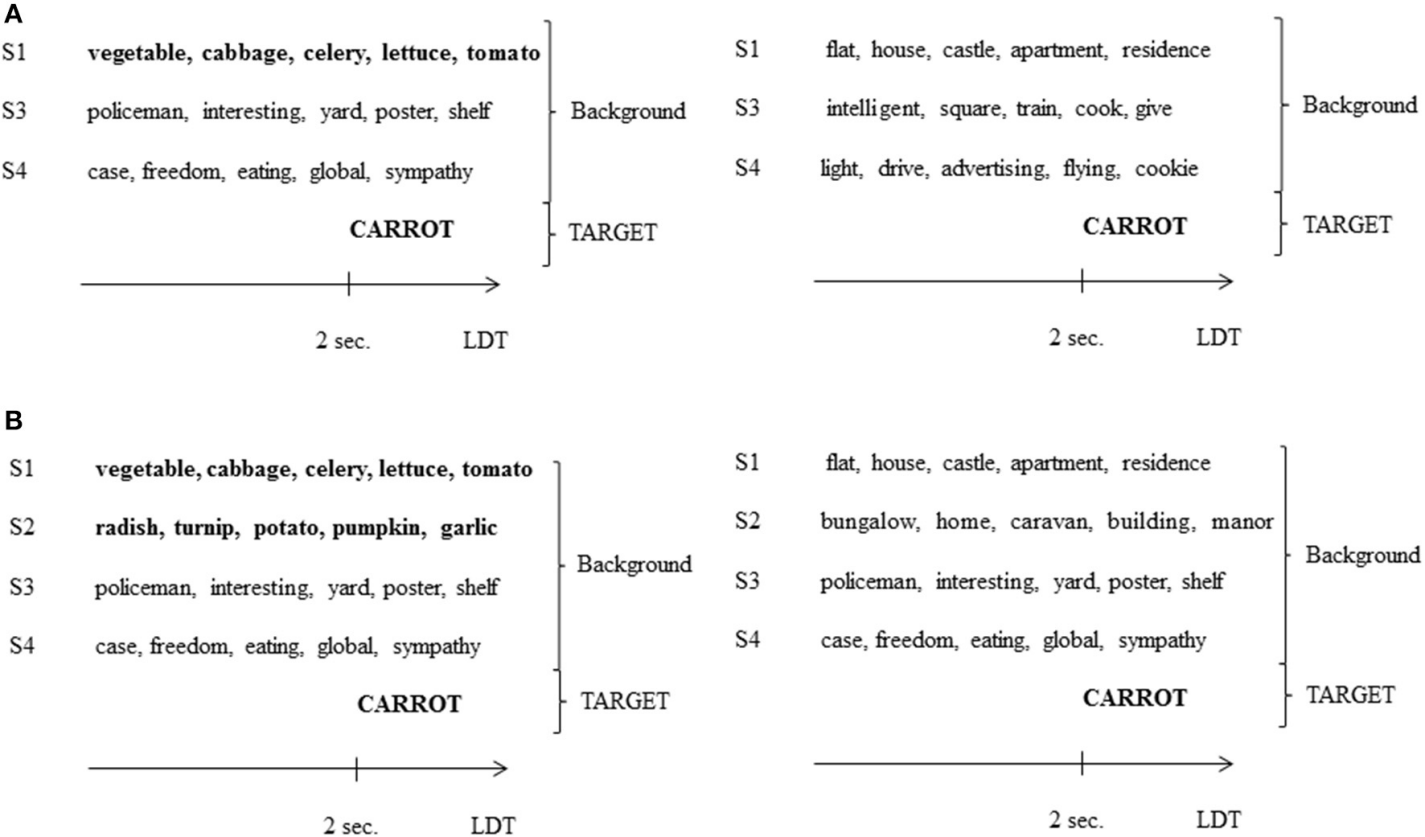
**(A)** Example of a background in the 1SC/2SI condition of Experiment 3, presented with a semantically related target word (left; related condition) or not (right; unrelated condition). S4, speaker 4 (see the legend of Figures 1, 2 for other abbreviations). **(B)** Example of a background in the 2SC/2SI condition of Experiment 3, presented with a semantically related target word (left; related condition) or not (right; unrelated condition).

**Figure 4:**
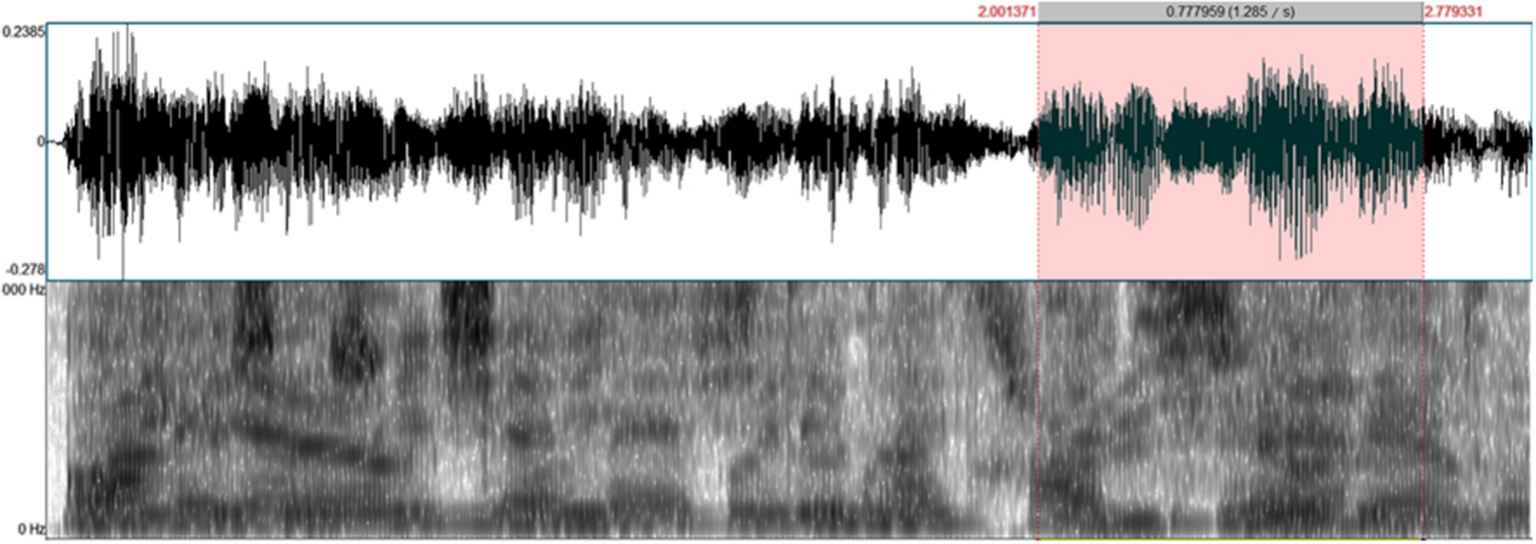
**Example of a spectrogram and a wave form of a 2SC/2SI condition stimulus**. Red part corresponds to the occurrence of the target word.

The second SI voice (S4) was recorded by a French native female speaker (age = 23), using the same procedure as in previous experiments. S1, S2, and S3's auditory sequences were mixed with S4's. Targets used in Experiment 2 (recorded by TS2) were embedded in the backgrounds 2 s after their beginning start (see Figures 3, 4).

#### Recognition post-test

A recognition test was devised to test whether participants could recognize words previously heard during the experiment. Fifty words were presented to participants after the main experiment on a sheet of paper, 20 had been previously presented in the backgrounds, whereas 30 were new words not used as stimuli in the experiment. A list of new words was generated (*M*_lexical frequency_ = 23.15, *SD* = 48.29), and their lexical frequency did not significantly differ from that of previously heard words (*M*_lexical frequency_ = 18.47, *SD* = 20.61; *F* < 1).

#### Procedure

The same procedure was used as in Experiments 1 and 2. At the end of the experiment, a post-test was given to participants, instructing them to decide (i.e., write down) whether they had heard the given word during the experiment. They were asked to do the best they could but not to think about it too much, and to simply answer what they thought was right.

### Results

#### Test

The same statistical analyses as in Experiments 1 and 2 were performed by participants (*F*_1_) and by items (*F*_2_), with RTs (in ms) and ERs for target word identification as dependent variables. We included Number of Voices in the background (3 voices, 1SC/2SI condition or 4 voices, 2SC/2SI condition) and Semantic Link between prime and target (related or unrelated) as within-subjects factors. Target words answered correctly by less than half of participants were excluded from analyses (*CHIGNON, EPAULE*, and *HIBOU*, “bun, shoulder, owl”). In total, 16.8% of data were excluded from RTs analysis because of errors (15%) or extreme values (1.8%). Means and SDs for RTs and ERs are summarized in Table 3.

**Table 3:** **Means and SDs of RTs and ERs depending on the number of voices in the background and the semantic link between prime and target in Experiment 3**.

		**1SC/2SI**		**2SC/2SI**	
		**Related**	**Unrelated**	**Related**	**Unrelated**
RT (ms)	Mean	924	928	928	953
	*SD*	157	158	156	157
ER (%)	Mean	7.03	11.1	13.00	15.27
	*SD*	7.30	9.64	11.69	7.88

*1SC/2SI, 1 SC voice and 2 SI voices condition; 2SC/2SI, 2 SC voices and 2 SI voices condition*.

The analysis by participants revealed a significant main effect of the Number of Voices on RTs [*F*_1(1, 23)_ = 4.01, *p* = 0.05; *F*_2_ < 1]. Participants more quickly identified target words if backgrounds were composed of 3 voices (1SC/2SI condition; *M* = 925 ms, *SD* = 155) than if they were composed of 4 voices (2SC/2SI condition; *M* = 941 ms, *SD* = 155). The ERs analysis also showed that participants responded significantly more accurately [*F*_1(1, 23)_ = 6.19, *p* < 0.05; *F*_2(1, 44)_ = 5.80, *p* < 0.05] in the 1SC/2SI condition (*M* = 10.02%, *SD* = 10.10) than in the 2SC/2SI condition (*M* = 13.21%, *SD* = 8.95). The main effect of Semantic Link was not significant for either RTs [*F*_1(1, 23)_ = 1.51, *n.s*.; *F*_2(1, 44)_ = 3.21, *n.s*.] or ERs [*F*_1(1, 23)_ = 3.19, *n.s*.; *F*_2(1, 44)_ = 2.92, *n.s*.]. No significant interaction emerged between the 2 factors for RTs [*F*_1(1, 23)_ = 1.39, *n.s*.; *F*_2(1, 44)_ = 1.10, *n.s*.] and ERs (*F*_1_ < 1; *F*_2_ < 1). These last results indicate that participants performed better in the lexical decision task, both in terms of speed and accuracy, if the backgrounds were composed of 3 voices rather than 4 voices. This finding highlights the impact of masking on target recognition. However, no significant semantic priming effect was observed in these conditions, suggesting that informational masking from 1SC/2SI and 2SC/2SI backgrounds was so efficient that it prevented semantic processing of the prime, which could therefore not affect target word identification. Additionally, in the 2SC/2SI condition, energetic masking was more important; this factor might also explain the important masking of prime and explain the lack of semantic priming.

#### Post-test

Analysis of the participants' answers showed a mean ER of 44.5% (*SD* = 7) and *d*' = 0.26 (*SD* = 0.5). Although these results are close to chance, both were significant results, as shown by a one-sample *t*-test (ER *p* < 0.01; *d*' *p* < 0.05). No significant correlation was found between *d*' and priming effect (*r* = −0.39, *n.s*.). This finding implies that participants heard some background words, but those words were not used to improve performances. Additionally, a repeated measure ANOVA was performed with ERs as the dependent variable and number of Voices (3 or 4) in the background and Semantic Link between presented word and target as a within subjects factor. Neither the effect of the Number of Voices nor the effect of Semantic Link were significant (*F*_Number of Voices_ < 1; *F*_Semantic Link_ < 1).

### Discussion

In this third experiment, target word identification was again disturbed by the increase in the number of voices in the backgrounds, confirming that masking is more efficient in the 4-voice than in the 3-voice condition. Disappearance of the semantic priming effect also suggests that the number of SC voices compared to the number of SI voices was too small. To compare the data of the 3 experiments, we considered the ratio of SC voices over the total number of voices. Therefore, the ratio of SC/total voices is 1 in 1SC and 2SC conditions; ratio 2/3 in 2SC/1SI; ratio 1/2 in 1SC/2SI and 2SC/2SI; and ratio 1/3 in 1SC/2SI. An ANCOVA on all data using the number of voices as the independent variable and the ratio of SC voices/total voices as covariate on priming effect confirmed this hypothesis (effect of ratio: *p* < 0.05): when the ratio was too low, SC voices were not salient enough for participants to perform semantic processing. In a post-test, participants were asked to perform a recognition task immediately after the experiment. Participants scored significantly better than chance at the recognition test, showing that at least some words in the background were identified. This finding is consistent with the results by Hoen et al. (2007) who showed that in a transcription task, with up to 4 voices in the background, participants gave words from the background as responses instead of target words. Overall Experiment 3 showed that participants were unable to process the prime at a semantic level (i.e., no priming effect was observed) although the post-test results suggest that they hear it sufficiently to recognize it in a recognition post-test. This finding suggests that a word can be heard and implicitly encoded without being sufficiently deeply processed to elicit semantic priming.

## *Post-hoc* analyses

**Figure 5:**
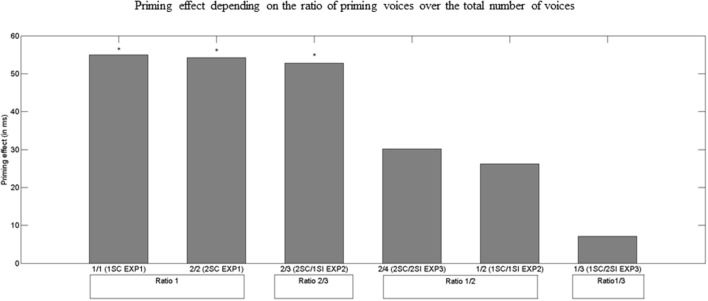
**Semantic priming effect (i.e., difference between RTs for unrelated and related targets; in ms) depending on the ratio of SC voices in the background over the total number of voices**. 1SC EXP1, 1SC condition in Experiment 1 (1 SC voice; no SI voice); 2SC EXP1, 2SC condition in Experiment 1 (2 SC voices; no SI voice); 2SC/1SI EXP2, 2SC/1SI condition in Experiment 2 (2 SC voices; 1 SI voice); 1SC/1SI EXP2, 1SC/1SI condition in Experiment 2 (1 SC voice; 1 SI voice); and 2SC/2SI EXP3, 2SC/2SI condition in Experiment 3 (2 SC voices; 2 SI voices); 1SC/2SI EXP3, 1SC/2SI condition in Experiment 3 (1 SC voice; 2 SI voices).

To better analyze the impact of the SC/total voices ratio, we conducted *post-hoc* analyses. A HSD Tukey test showed that up to 4 voices in the background if the ratio of SC/total voices was inferior to 1/2, there was no significant semantic priming effect (Experiment 2. 1SC/1SI; Experiment 3. 1SC/2SI and 2SC/2SI). However, if the ratio was superior to 1/2, semantic priming was significant (Experiment 1: 1SC, *p* < 0.05 Cohen's *d* = 0.31; 2SC, *p* < 0.05, Cohen's *d* = 0.35; Experiment 2. 2SC/1SI, *p* < 0.05, Cohen's *d* = 0.31; cf Figure 5 and Table 4).

**Table 4:** **Mean priming effect (in ms), SD, and *p*-value for each ratio**.

**Ratio**	**1**		**2/3**	**1/2**		**1/3**
**Condition**	**1SC**	**2SC**	**2SC/1SI**	**1SC/1SI**	**2SC/2SI**	**1SC/2SI**
	**(EXP1)**	**(EXP1)**	**(EXP2)**	**(EXP2)**	**(EXP3)**	**(EXP3)**
Mean	55	56	55	25	26	4
*SD*	70	119	110	88	83	65
*P*	0.03^*^	0.03^*^	0.007^*^	0.88	0.21	0.99

* *Indicates significant priming. The ratio is expressed in terms of the number of SC voices over the total number of voices in the background: 1, 1 SC voice or 2 SC voices; 2/3, 2 SC voices, 1 SI voice; 1/2, 1 SC voice, 1 SI voice or 2 SC voices, 2 SI voices; 1/3, 1 SC voice, 2 SI voices*.

## General discussion

### Summary

The aim of this study was to investigate to what extent the semantic content of the multi-talker babble could interfere with the processing of targets in a cocktail party situation. In the three reported experiments, participants were required to perform a lexical decision task on a target item embedded in a multi-talker background. The backgrounds were composed of 1 or 2 SC voices pronouncing words that shared semantic features with each other and could be semantically related to the target or not. The ratio of SC voices over the total number of voices in the background was varied across experiments. In Experiment 1, ratios were 1/1 and 2/2 (i.e., only SC voices were presented in the background). In Experiments 2 and 3, 1 and 2 SI voices, respectively, that pronounced semantically unrelated words, were added to backgrounds to decrease the intelligibility of the SC voices, which acted as the prime. The ratio of SC/total voices in Experiment 2 was therefore 1/2 and 2/3 and in Experiment 3 it was 1/3 and 2/4.

The main effect of the Number of Voices was significant in each experiment; participants responded faster and more accurately to target words if a smaller number of voices composed the backgrounds. This delayed response time with increasing number of voices is a well-known phenomenon (Brungart et al., 2001; Boulenger et al., 2010). As the number of voices increases, energetic masking is enhanced so that the signal is saturated, and target items are consequently more difficult to process. In addition to physical masking, more information is perceived and must be processed (i.e., informational masking), leading to slower response times.

Overall the main effect of Semantic Link between prime and target seems to be an all-or-none phenomenon. The semantic priming effect could have decreased with an increasing number of voices in the background, but our results suggest this effect does not occur, as no interaction between the Number of Voices and the Semantic Link was observed in any of the three experiments. The main effect of Semantic Link depends on the ratio of SC voices over the total number of voices in the background. A semantic priming effect emerged in our experiments spanning from 1 to 4 voices in the background but only if the number of SC voices was higher than the number of SI voices. This ratio of SC voices is interesting because it highlights the necessity of prime saliency for semantic processing to occur; it also gives an objective measure of this prime saliency across experiments. This finding suggests that informational masking does occur at the semantic level but only if the prime is sufficiently salient. If informational and energetic masking had the same role, a significant semantic priming effect would appear in conditions containing 3 voices (i.e., Experiment 2 2SC/1SI and Experiment 3 1SC/2SI). However, this effect did not occur; we therefore conclude that informational masking can be semantic only if it is sufficiently salient to be used to increase performance.

### Lexical processing without semantic activation

Our results suggest that semantic processing in cocktail party situation is not automatic. In fact, it seems that in challenging listening situations, one can hear and activate a mental representation of a word without deeply processing it at a semantic level. Background words can be semantically processed with up to 3 voices (2SC/1SI, Experiment 2); however no priming effect emerged in Experiment 3, despite the recognition post-test's results suggesting that primes were heard and recognized. This finding is consistent with the way the language system has been modeled; most models of word processing, both in the auditory and the visual modalities, suggest a distinction between lexical and semantic levels of processing (Marslen-Wilson and Welsh, 1978; McClelland and Elman, 1986; Grainger and Holcomb, 2009). If one considers that these processes are independent stages of word recognition, it seems reasonable that one of these stages can be reached (i.e., lexical) without strongly activating the deepest stage (i.e., semantic).

Many studies have highlighted the automaticity of semantic processing using masked semantic priming (Naccache and Dehaene, 2001; Klauer et al., 2007; Kouider and Dehaene, 2009; Spruyt et al., 2012). However, masked semantic priming is only found in very specific conditions in the visual modality and with semantic categorization tasks (see Van den Bussche et al., 2009, for a review). One can therefore assume that semantic activation remains superficial in masked priming paradigm, and only activates very close concepts such as superordinates, which is enough to create a priming effect in semantic categorization tasks. In our experiments however, a deeper semantic processing was necessary. For example, in the semantic field of birds, the SC voice pronounced: *corbeau, rossignol, cage, voler, nid* (“craw, nightingale, cage, fly, nest”) and *PIGEON* (“pigeon”) was the target word. In a condition of high intelligibility, these words primed the target word; however, with decreased intelligibility, if the participants only heard the word “cage,” this word may have activated its superordinate (e.g., “object”) but not its associates such as “bird.” Consequently, it may not have primed “pigeon.” The absence of a semantic priming effect in addition to the decrease in intelligibility seems to show that greater difficulty in processing auditory signals at a superficial level, causes decreased processing at a (higher) linguistic level.

Consistent with our results, and using an auditory masked priming paradigm, Kouider and Dupoux (2005) demonstrated lexical access without semantic priming. In their experiments, prime was a time-compressed word embedded in a masker composed of time reversed and compressed words. They manipulated the compression rate of the prime to vary its intelligibility. If the prime was not intelligible, they found a significant repetition priming effect on words but not non-words suggesting that this effect “involved a lexical activation of abstract word form.” In the same condition of prime compression, they did not find any semantic priming effect. This finding would therefore suggest a dissociation between lexical and semantic processing.

### Cognitive load

Overall our results are compatible with the idea of cognitive load, in which simultaneously processed information and interactions can either under-load or overload the finite amount of processing capacities. As shown by our results, it is more difficult to process items with more voices in the background. This finding might be partly due to the high perceptual load and because the target item was embedded in backgrounds that could not be completely ignored (Lavie, 1995, 2005). Processing words in the background might have been particularly cognitively effortful and, semantic processing of a word can be delayed and even prevented if participants have to perform an additional task (i.e., high cognitive load, Hohlfeld et al., 2004; Hohlfeld and Sommer, 2005; Van Petten, 2014). In Experiment 3 we argue that some background words were heard but not deeply semantically processed because it was both very demanding and irrelevant to perform the task. In Experiment 2, 2SC/1SI background words were also very difficult to hear and process (as in Experiment 3 1SC/2SI); however, they were semantically processed as revealed by the semantic priming effect observed. As SC voices were more salient in Experiment 2 2SC/1SI than in Experiment 3 1SC/2SI, participants might have heard more related words and one could argue that semantic priming in our study in fact relied on the chance for participants to hear a SC word. Although this hypothesis is interesting, it does not seem sufficient to explain our results. Indeed, according to this hypothesis, a priming effect should have appeared also in Experiment 3 where participants, in line with previous results from the literature (Brungart, 2001; Hoen et al., 2007), recognized SC words presented in the post-test. Given the overall results of our experiments, we argue that if the ratio of SC/total voices was <1/2, participants heard some SC words, but, because of high cognitive load (i.e., intelligibility was low and they focused on the target item) these words were not processed sufficiently deeply to lead to semantic priming (a similar dissociation between lexical and semantic processing was also found by Kouider and Dupoux, 2005). However, if the ratio was >1/2, SC words were salient, and participants thus, processed them sufficiently to improve their performance. As this interpretation relies on the post-test effect which is quite small, although significant, more experiments should be performed. For example, using only SC voices and degrading intelligibility by adding noise or filtering the signal instead of adding SI voice would be a good way to test this hypothesis in future studies.

Our results suggest that the increased cognitive load necessary to reconstruct the degraded signal reduced available resources for higher-level processes. This claim is consistent with the Effortfulness Hypothesis (Rabbitt, 1968) that states degraded signals require allocating many cognitive resources to formal processes (i.e., orthographic or phonological), leaving less available cognitive resources to perform higher-level processes (e.g., lexical). It has been shown that hearing-impaired participants are less accurate at recalling previously heard final sentence words than their control peers (Pichora-Fuller et al., 1995). The underlying assumption is that for hearing-impaired participants, the auditory signal is highly degraded and therefore demands more cognitive resources to be formally (i.e., phonologically) processed. As studies usually use recall tasks of lists of unrelated words or digits (Surprenant, 1999; Murphy et al., 2000; Wingfield et al., 2005), verbal working memory only relies on the phonological loop (Baddeley and Hitch, 1974; Baddeley, 2000) and only involves formal processes. Our results showed that higher levels of processing such as semantic activation may be specifically impacted by signal degradation.

Recently, semantic and syntactic integration difficulties if a signal is degraded have been reported in the visual modality (Gao et al., 2011, 2012). Experiments conducted by Gao et al. (2011) using visual noise (i.e., pixel's brightness variation) showed that if participants allocate more resources to formal processes, semantic integration is affected. Indeed, after reading an entire text, participants were worse at recalling the main proposition in the noisy condition. Altogether, these findings suggest that the availability of cognitive resources is involved at various levels during language processing. Whereas previous studies have shown that noise impairs memory systems, our study provides evidence that semantic activation is linked to cognitive resources, independently of memory.

## Conclusion

This study explored the semantic nature of informational masking in a cocktail party situation. The results of three behavioral studies reveal that the emergence of semantic priming effects relies on prime intelligibility and saliency. These findings question the assumption that signal degradation has no effect on speech processing if target signals can be recognized. The results reveal that high-level processes, such as semantic processing, might not be as automatic as previously thought but are subjected to the limits of cognitive resources. Our study also demonstrates how the cocktail party situation can be used to study the automaticity of linguistic processes.

## Conflict of interest statement

The authors declare that the research was conducted in the absence of any commercial or financial relationships that could be construed as a potential conflict of interest.
