# Supplementary material for: Multi-talker background and semantic priming effect
Source: Front Hum Neurosci. 2014 Oct 31;8:878. doi: 10.3389/fnhum.2014.00878 (PMC4215827; doi:10.3389/fnhum.2014.00878)
Supplement: Supplementary file 1 [file DataSheet1.DOCX]

**Annexes**

|  | **PRIME** | **TARGET** |  |  | **PRIME** | **TARGET** |
| --- | --- | --- | --- | --- | --- | --- |
|  |  |  |  |  |  |  |
| S1 | corbeau | **pigeon** |  | S1 | baigner | **serviette** |
|  | voler |  |  |  | sable |  |
|  | nid |  |  |  | pelle |  |
|  | hirondelle |  |  |  | seau |  |
|  | merle |  |  |  | plage |  |
|  |  |  |  |  |  |  |
| S2 | cage |  |  | S2 | bronzer |  |
|  | moineau |  |  |  | transat |  |
|  | oiseau |  |  |  | solaire |  |
|  | roucouler |  |  |  | touriste |  |
|  | volière |  |  |  | vacances |  |
|  |  |  |  |  |  |  |
| S1 | informatique | **écran** |  | S1 | vin | **champagne** |
|  | clavier |  |  |  | célébrer |  |
|  | ordinateur |  |  |  | trinquer |  |
|  | enceinte |  |  |  | alcool |  |
|  | programme |  |  |  | cave |  |
|  |  |  |  |  |  |  |
| S2 | cliquer |  |  | S2 | boire |  |
|  | fichier |  |  |  | vodka |  |
|  | connexion |  |  |  | sommelier |  |
|  | imprimante |  |  |  | millésime |  |
|  | réseau |  |  |  | cocktail |  |
|  |  |  |  |  |  |  |
| S1 | vache | **cochon** |  | S1 | sculpteur | **poète** |
|  | élever |  |  |  | métier |  |
|  | poule |  |  |  | enseignant |  |
|  | chèvre |  |  |  | maçon |  |
|  | tracteur |  |  |  | peintre |  |
|  |  |  |  |  |  |  |
| S2 | âne |  |  | S2 | exercer |  |
|  | lapin |  |  |  | cinéaste |  |
|  | fermier |  |  |  | acteur |  |
|  | traire |  |  |  | plombier |  |
|  | étable |  |  |  | médecin |  |

|  | **PRIME** | **TARGET** |  |  | **PRIME** | **TARGET** |  |
| --- | --- | --- | --- | --- | --- | --- | --- |
|  |  |  |  |  |  |  |  |
| S1 | tourelle | **château** |  | S1 | planer | **hibou** |  |
|  | armure |  |  |  | chouette |  |  |
|  | roi |  |  |  | serre |  |  |
|  | défendre |  |  |  | falaise |  |  |
|  | régner |  |  |  | aigle |  |  |
|  |  |  |  |  |  |  |  |
| S2 | donjon |  |  | S2 | fauconnier |  |  |
|  | palefrenier |  |  |  | rapace |  |  |
|  | pont |  |  |  | condor |  |  |
|  | douve |  |  |  | vautour |  |  |
|  | cachot |  |  |  | nocturne |  |  |
|  |  |  |  |  |  |  |  |
| S1 | souche | **racine** |  | S1 | dompter | **morsure** |  |
|  | écorce |  |  |  | jaguar |  |  |
|  | sève |  |  |  | sauvage |  |  |
|  | feuille |  |  |  | rapide |  |  |
|  | tronc |  |  |  | léopard |  |  |
|  |  |  |  |  |  |  |  |
| S2 | branche |  |  | S2 | puma |  |  |
|  | élaguer |  |  |  | traquer |  |  |
|  | nœud |  |  |  | proie |  |  |
|  | cime |  |  |  | panthère |  |  |
|  | résine |  |  |  | lynx |  |  |
|  |  |  |  |  |  |  |  |
| S1 | guichet | **billet** |  | S1 | créer | **artiste** |  |
|  | train |  |  |  | pinceau |  |  |
|  | gare |  |  |  | aquarelle |  |  |
|  | composter |  |  |  | chevalet |  |  |
|  | cheminot |  |  |  | toile |  |  |
|  |  |  |  |  |  |  |  |
| S2 | rail |  |  | S2 | peinture |  |  |
|  | contrôleur |  |  |  | gouache |  |  |
|  | wagon |  |  |  | tableau |  |  |
|  | terminus |  |  |  | crayonner |  |  |
|  | locomotive |  |  |  | atelier |  |  |

|  | **PRIME** | **TARGET** |  |  | **PRIME** | **TARGET** |  |
| --- | --- | --- | --- | --- | --- | --- | --- |
|  |  |  |  |  |  |  |  |
| S1 | pull | **gilet** |  | S1 | minute | **seconde** |  |
|  | chemise |  |  |  | trimestre |  |  |
|  | vêtir |  |  |  | heure |  |  |
|  | débardeur |  |  |  | chronométrer | |  |
|  | chandail |  |  |  | lundi |  |  |
|  |  |  |  |  |  |  |  |
| S2 | chaussette |  |  | S2 | sablier |  |  |
|  | jupon |  |  |  | attendre |  |  |
|  | bermuda |  |  |  | date |  |  |
|  | habiller |  |  |  | année |  |  |
|  | pantalon |  |  |  | calendrier |  |  |
|  |  |  |  |  |  |  |  |
| S1 | détenu | **prison** |  | S1 | pêcher | **filet** |  |
|  | arrêter |  |  |  | ligne |  |  |
|  | tribunal |  |  |  | canne |  |  |
|  | parloir |  |  |  | ferrer |  |  |
|  | juger |  |  |  | bouchon |  |  |
|  |  |  |  |  |  |  |  |
| S2 | cellule |  |  | S2 | hameçon |  |  |
|  | réclusion |  |  |  | vers |  |  |
|  | police |  |  |  | épuisette |  |  |
|  | avocat |  |  |  | chalutier |  |  |
|  | criminel |  |  |  | moulinet |  |  |
|  |  |  |  |  |  |  |  |
| S1 | bonnet | **foulard** |  | S1 | cil | **pupille** |  |
|  | chapeau |  |  |  | iris |  |  |
|  | béret |  |  |  | mascara |  |  |
|  | cagoule |  |  |  | lunette |  |  |
|  | sombrero |  |  |  | myopie |  |  |
|  |  |  |  |  |  |  |  |
| S2 | chapelier |  |  | S2 | loucher |  |  |
|  | couvrir |  |  |  | cerne |  |  |
|  | casque |  |  |  | sourcil |  |  |
|  | visière |  |  |  | maquiller |  |  |
|  | képi |  |  |  | lentille |  |  |

|  | **PRIME** | **TARGET** |  |  | **PRIME** | **TARGET** |
| --- | --- | --- | --- | --- | --- | --- |
|  |  |  |  |  |  |  |
| S1 | casier | **buffet** |  | S1 | curry | **cannelle** |
|  | coffre |  |  |  | épicer |  |
|  | trier |  |  |  | piment |  |
|  | commode |  |  |  | oriental |  |
|  | placard |  |  |  | muscade |  |
|  |  |  |  |  |  |  |
| S2 | meuble |  |  | S2 | gingembre |  |
|  | plier |  |  |  | poivre |  |
|  | vêtement |  |  |  | coriandre |  |
|  | armoire |  |  |  | arôme |  |
|  | bureau |  |  |  | relever |  |
|  |  |  |  |  |  |  |
| S1 | robinet | **baignoire** |  | S1 | verre | **couteau** |
|  | lavabo |  |  |  | manger |  |
|  | évier |  |  |  | nappe |  |
|  | tremper |  |  |  | attabler |  |
|  | shampooing |  |  |  | couvert |  |
|  |  |  |  |  |  |  |
| S2 | laver |  |  | S2 | cuiller |  |
|  | douche |  |  |  | fourchette |  |
|  | Savon |  |  |  | servir |  |
|  | peignoir |  |  |  | table |  |
|  | mouiller |  |  |  | repas |  |
|  |  |  |  |  |  |  |
| S1 | carreau | **rideau** |  | S1 | chêne | **platane** |
|  | ouverture |  |  |  | pommier |  |
|  | aérer |  |  |  | hêtre |  |
|  | lucarne |  |  |  | merisier |  |
|  | baie |  |  |  | scier |  |
|  |  |  |  |  |  |  |
| S2 | vitre |  |  | S2 | érable |  |
|  | fenêtre |  |  |  | oranger |  |
|  | balcon |  |  |  | fleurir |  |
|  | persienne |  |  |  | frêne |  |
|  | store |  |  |  | verger |  |

|  | **PRIME** | **TARGET** |  |  | **PRIME** | **TARGET** |
| --- | --- | --- | --- | --- | --- | --- |
|  |  |  |  |  |  |  |
| S1 | carreler | **parquet** |  | S1 | marmite | **cocotte** |
|  | lino |  |  |  | sauteuse |  |
|  | dalle |  |  |  | poêlon |  |
|  | faïence |  |  |  | casserole |  |
|  | artisan |  |  |  | cuisson |  |
|  |  |  |  |  |  |  |
| S2 | joint |  |  | S2 | mijoter |  |
|  | mosaïque |  |  |  | ébullition |  |
|  | plancher |  |  |  | cuisiner |  |
|  | béton |  |  |  | cuire |  |
|  | moquette |  |  |  | gazinière |  |
|  |  |  |  |  |  |  |
| S1 | bus | **métro** |  | S1 | potager | **carotte** |
|  | transiter |  |  |  | légume |  |
|  | commun |  |  |  | arrosage |  |
|  | horaire |  |  |  | fertilisant |  |
|  | arrêt |  |  |  | limace |  |
|  |  |  |  |  |  |  |
| S2 | navette |  |  | S2 | terreau |  |
|  | terminus |  |  |  | jardinage |  |
|  | tramway |  |  |  | bêcher |  |
|  | voyager |  |  |  | céleri |  |
|  | car |  |  |  | navet |  |
|  |  |  |  |  |  |  |
| S1 | basket | **lacet** |  | S1 | caniche | **berger** |
|  | pantoufle |  |  |  | basset |  |
|  | botte |  |  |  | chihuahua |  |
|  | soulier |  |  |  | labrador |  |
|  | chausse-pied |  |  |  | bichon |  |
|  |  |  |  |  |  |  |
| S2 | escarpin |  |  | S2 | chien |  |
|  | sabot |  |  |  | lévrier |  |
|  | chausson |  |  |  | pédigrée |  |
|  | marcher |  |  |  | épagneul |  |
|  | ballerine |  |  |  | Cocker |  |

|  | **PRIME** | **TARGET** |  |  | **PRIME** | **TARGET** |
| --- | --- | --- | --- | --- | --- | --- |
|  |  |  |  |  |  |  |
| S1 | doigt | **poignet** |  | S1 | guitare | **violon** |
|  | ongle |  |  |  | mandoline |  |
|  | attraper |  |  |  | contrebasse |  |
|  | manucure |  |  |  | mélodie |  |
|  | tenir |  |  |  | orchestre |  |
|  |  |  |  |  |  |  |
| S2 | pouce |  |  | S2 | banjo |  |
|  | manipuler |  |  |  | harpe |  |
|  | phalange |  |  |  | trompette |  |
|  | bague |  |  |  | symphonie |  |
|  | main |  |  |  | interpréter |  |
|  |  |  |  |  |  |  |
| S1 | nez | **figure** |  | S1 | cuisiner | **gigot** |
|  | joues |  |  |  | potée |  |
|  | front |  |  |  | dégustation |  |
|  | visagiste |  |  |  | viande |  |
|  | grimacer |  |  |  | gourmet |  |
|  |  |  |  |  |  |  |
| S2 | farder |  |  | S2 | barbecue |  |
|  | oreille |  |  |  | rôti |  |
|  | pommette |  |  |  | saucisse |  |
|  | bouche |  |  |  | fumé |  |
|  | menton |  |  |  | grillade |  |
|  |  |  |  |  |  |  |
| S1 | huitre | **crevette** |  | S1 | grandir | **épaule** |
|  | moule |  |  |  | cuisse |  |
|  | bulot |  |  |  | coude |  |
|  | décortiquer |  |  |  | hanche |  |
|  | palourde |  |  |  | bras |  |
|  |  |  |  |  |  |  |
| S2 | praire |  |  | S2 | genou |  |
|  | homard |  |  |  | pied |  |
|  | langoustine |  |  |  | cheville |  |
|  | crabe |  |  |  | poignet |  |
|  | gambas |  |  |  | jambe |  |

|  | **PRIME** | **TARGET** |  |  | **PRIME** | **TARGET** |
| --- | --- | --- | --- | --- | --- | --- |
|  |  |  |  |  |  |  |
| S1 | corail | **trésor** |  | S1 | prêter | **salaire** |
|  | masque |  |  |  | découvert |  |
|  | plonger |  |  |  | banquier |  |
|  | épave |  |  |  | dépenser |  |
|  | sable |  |  |  | épargne |  |
|  |  |  |  |  |  |  |
| S2 | algue |  |  | S2 | crédit |  |
|  | galet |  |  |  | emprunter |  |
|  | tuba |  |  |  | banque |  |
|  | récif |  |  |  | débit |  |
|  | palme |  |  |  | hypothèque |  |
|  |  |  |  |  |  |  |
| S1 | plomb | **acier** |  | S1 | terrier | **raton** |
|  | fer |  |  |  | furet |  |
|  | cuivre |  |  |  | ronger |  |
|  | souder |  |  |  | souris |  |
|  | chaudronnier |  |  |  | écureuil |  |
|  |  |  |  |  |  |  |
| S2 | bronze |  |  | S2 | hamster |  |
|  | forgeron |  |  |  | mulot |  |
|  | métal |  |  |  | cobaye |  |
|  | zinc |  |  |  | gerbille |  |
|  | étain |  |  |  | loir |  |
|  |  |  |  |  |  |  |
| S1 | bague | **collier** |  | S1 | astronaute | **planète** |
|  | chaine |  |  |  | télescope |  |
|  | montre |  |  |  | constellation |  |
|  | parer |  |  |  | fusée |  |
|  | médaille |  |  |  | orbite |  |
|  |  |  |  |  |  |  |
| S2 | pendentif |  |  | S2 | étoile |  |
|  | bijou |  |  |  | comète |  |
|  | chevalière |  |  |  | galaxie |  |
|  | orner |  |  |  | astre |  |
|  | diadème |  |  |  | alunir |  |

|  | **PRIME** | **TARGET** |  |  | **PRIME** | **TARGET** |
| --- | --- | --- | --- | --- | --- | --- |
|  |  |  |  |  |  |  |
| S1 | ramper | **vipère** |  | S1 | volant | **essence** |
|  | crocodile |  |  |  | capot |  |
|  | serpent |  |  |  | banquette |  |
|  | caïman |  |  |  | conduire |  |
|  | reptile |  |  |  | circulation |  |
|  |  |  |  |  |  |  |
| S2 | boa |  |  | S2 | caravane |  |
|  | iguane |  |  |  | rouler |  |
|  | python |  |  |  | voiture |  |
|  | lézards |  |  |  | autoroute |  |
|  | tortue |  |  |  | frein |  |
|  |  |  |  |  |  |  |
| S1 | cheminée | **foyer** |  | S1 | aiguille | **tissu** |
|  | poêle |  |  |  | fil |  |
|  | foyer |  |  |  | coudre |  |
|  | cendre |  |  |  | ourlet |  |
|  | ramoner |  |  |  | patron |  |
|  |  |  |  |  |  |  |
| S2 | allumette |  |  | S2 | broderie |  |
|  | barbecue |  |  |  | enfiler |  |
|  | flamber |  |  |  | tricoter |  |
|  | suie |  |  |  | bouton |  |
|  | étincelle |  |  |  | dé |  |
|  |  |  |  |  |  |  |
| S1 | teindre | **chignon** |  | S1 | croix | **prière** |
|  | peigne |  |  |  | chapelle |  |
|  | brosse |  |  |  | crypte |  |
|  | pince |  |  |  | église |  |
|  | bigoudis |  |  |  | messe |  |
|  |  |  |  |  |  |  |
| S2 | cheveux |  |  | S2 | curé |  |
|  | tresse |  |  |  | clocher |  |
|  | coiffer |  |  |  | autel |  |
|  | rincer |  |  |  | bible |  |
|  | démêler |  |  |  | évangile |  |

|  | **PRIME** | **TARGET** |  |  | **PRIME** | **TARGET** |
| --- | --- | --- | --- | --- | --- | --- |
|  |  |  |  |  |  |  |
| S1 | tactile | **pormou** |  | S1 | couche | **lofite** |
|  | sentir |  |  |  | bavoir |  |
|  | gouter |  |  |  | sucette |  |
|  | senteur |  |  |  | berceuse |  |
|  | écoute |  |  |  | poussette |  |
|  |  |  |  |  |  |  |
| S2 | odorat |  |  | S2 | pleurer |  |
|  | texture |  |  |  | lange |  |
|  | gout |  |  |  | tétine |  |
|  | saveur |  |  |  | veilleuse |  |
|  | sensation |  |  |  | pouponner |  |
|  |  |  |  |  |  |  |
| S1 | noël | **humel** |  | S1 | caisse | **roteau** |
|  | avent |  |  |  | rayons |  |
|  | offrir |  |  |  | panier |  |
|  | fêter |  |  |  | supermarché |  |
|  | dinde |  |  |  | dépenser |  |
|  |  |  |  |  |  |  |
| S2 | décorer |  |  | S2 | payer |  |
|  | lumière |  |  |  | shopping |  |
|  | trêve |  |  |  | caddie |  |
|  | sapin |  |  |  | achat |  |
|  | nativité |  |  |  | enseigne |  |
|  |  |  |  |  |  |  |
| S1 | coaguler | **tinate** |  | S1 | voile | **léquente** |
|  | vaccin |  |  |  | laine |  |
|  | seringue |  |  |  | couper |  |
|  | piquer |  |  |  | tisser |  |
|  | garrot |  |  |  | chute |  |
|  |  |  |  |  |  |  |
| S2 | allergie |  |  | S2 | filer |  |
|  | globule |  |  |  | popeline |  |
|  | rouge |  |  |  | Tulle |  |
|  | artère |  |  |  | coton |  |
|  | veine |  |  |  | épingler |  |

|  | **PRIME** | **TARGET** |  |  | **PRIME** | **TARGET** |
| --- | --- | --- | --- | --- | --- | --- |
|  |  |  |  |  |  |  |
| S1 | dauphin | **tofant** |  | S1 | guidon | **contreau** |
|  | cachalot |  |  |  | cycliste |  |
|  | sauter |  |  |  | vélo |  |
|  | phoque |  |  |  | course |  |
|  | mammifère |  |  |  | bicyclette |  |
|  |  |  |  |  |  |  |
| S2 | orque |  |  | S2 | pédaler |  |
|  | nageoire |  |  |  | roues |  |
|  | otarie |  |  |  | chaines |  |
|  | morse |  |  |  | béquille |  |
|  | plonger |  |  |  | vitesse |  |
|  |  |  |  |  |  |  |
| S1 | nettoyer | **molude** |  | S1 | cerveau | **vemune** |
|  | aspirateur |  |  |  | crâne |  |
|  | cirer |  |  |  | médical |  |
|  | propreté |  |  |  | soigner |  |
|  | serpillère |  |  |  | veine |  |
|  |  |  |  |  |  |  |
| S2 | ménage |  |  | S2 | estomac |  |
|  | balai |  |  |  | foie |  |
|  | torchon |  |  |  | chirurgien |  |
|  | chiffon |  |  |  | médicament |  |
|  | racloir |  |  |  | malade |  |
|  |  |  |  |  |  |  |
| S1 | selle | **ginou** |  | S1 | merlan | **trifeau** |
|  | dresser |  |  |  | dorade |  |
|  | manège |  |  |  | cabillaud |  |
|  | équitation |  |  |  | silure |  |
|  | cavalier |  |  |  | nager |  |
|  |  |  |  |  |  |  |
| S2 | stalle |  |  | S2 | saumon |  |
|  | renne |  |  |  | poisson |  |
|  | mors |  |  |  | perche |  |
|  | étriers |  |  |  | thon |  |
|  | cravache |  |  |  | sardine |  |

|  | **PRIME** | **TARGET** |  |  | **PRIME** | **TARGET** |
| --- | --- | --- | --- | --- | --- | --- |
|  |  |  |  |  |  |  |
| S1 | bateau | **oppard** |  | S1 | lèvre | **poceau** |
|  | dériver |  |  |  | plombage |  |
|  | gouvernail |  |  |  | saliver |  |
|  | matelot |  |  |  | parole |  |
|  | houle |  |  |  | croquer |  |
|  |  |  |  |  |  |  |
| S2 | naviguer |  |  | S2 | mordre |  |
|  | coque |  |  |  | dentiste |  |
|  | mat |  |  |  | déglutir |  |
|  | voile |  |  |  | étouffer |  |
|  | cabine |  |  |  | dent |  |
|  |  |  |  |  |  |  |
| S1 | réunion | **eimard** |  | S1 | siphon | **oqueaux** |
|  | cantine |  |  |  | plomberie |  |
|  | conseil |  |  |  | chaudière |  |
|  | employer |  |  |  | radiateur |  |
|  | patron |  |  |  | tuyau |  |
|  |  |  |  |  |  |  |
| S2 | adjoint |  |  | S2 | sanitaire |  |
|  | cadre |  |  |  | canalisation |  |
|  | collègue |  |  |  | fuite |  |
|  | secrétaire |  |  |  | clapet |  |
|  | congé |  |  |  | vidange |  |
|  |  |  |  |  |  |  |
| S1 | orner | **berté** |  | S1 | sommet | **gilot** |
|  | rubis |  |  |  | cordée |  |
|  | saphir |  |  |  | alpinisme |  |
|  | précieux |  |  |  | crevasse |  |
|  | caillou |  |  |  | grimper |  |
|  |  |  |  |  |  |  |
| S2 | opale |  |  | S2 | bouquetin |  |
|  | émeraude |  |  |  | chamois |  |
|  | grenat |  |  |  | pic |  |
|  | topaze |  |  |  | neige |  |
|  | pierre |  |  |  | randonnée |  |

|  | **PRIME** | **TARGET** |  |  | **PRIME** | **TARGET** |
| --- | --- | --- | --- | --- | --- | --- |
|  |  |  |  |  |  |  |
| S1 | oreiller | **encro** |  | S1 | collier | **touffi** |
|  | couette |  |  |  | chenil |  |
|  | allonger |  |  |  | laisse |  |
|  | couverture |  |  |  | pâtée |  |
|  | draps |  |  |  | niche |  |
|  |  |  |  |  |  |  |
| S2 | lit |  |  | S2 | croquette |  |
|  | sommier |  |  |  | promener |  |
|  | dormir |  |  |  | puce |  |
|  | sieste |  |  |  | aboiement |  |
|  | matelas |  |  |  | gueule |  |
|  |  |  |  |  |  |  |
| S1 | ballon | **ornece** |  | S1 | affiche | **poppou** |
|  | courir |  |  |  | cinéma |  |
|  | marquer |  |  |  | amusant |  |
|  | passe |  |  |  | découvrir |  |
|  | joueur |  |  |  | effrayant |  |
|  |  |  |  |  |  |  |
| S2 | rugby |  |  | S2 | fauteuil |  |
|  | lancer |  |  |  | regarder |  |
|  | terrain |  |  |  | nouveauté |  |
|  | touche |  |  |  | projection |  |
|  | mêlée |  |  |  | film |  |
|  |  |  |  |  |  |  |
| S1 | géométrie | **anquise** |  | S1 | génération | **plaro** |
|  | surface |  |  |  | proche |  |
|  | mesurer |  |  |  | parent |  |
|  | compas |  |  |  | frère |  |
|  | tracer |  |  |  | tante |  |
|  |  |  |  |  |  |  |
| S2 | équerre |  |  | S2 | épouser |  |
|  | rapporteur |  |  |  | ancêtre |  |
|  | polygone |  |  |  | oncle |  |
|  | volume |  |  |  | filiation |  |
|  | carré |  |  |  | neveu |  |

|  | **PRIME** | **TARGET** |  |  | **PRIME** | **TARGET** |
| --- | --- | --- | --- | --- | --- | --- |
|  |  |  |  |  |  |  |
| S1 | fraise | **nouson** |  | S1 | tableau | **umage** |
|  | groseille |  |  |  | cahier |  |
|  | cassis |  |  |  | trousse |  |
|  | croquer |  |  |  | règle |  |
|  | pomme |  |  |  | livre |  |
|  |  |  |  |  |  |  |
| S2 | banane |  |  | S2 | écolier |  |
|  | kiwi |  |  |  | leçon |  |
|  | compote |  |  |  | apprendre |  |
|  | confiture |  |  |  | craie |  |
|  | melon |  |  |  | gomme |  |
|  |  |  |  |  |  |  |
| S1 | opéra | **patoun** |  | S1 | émission | **naison** |
|  | déchiffrer |  |  |  | média |  |
|  | métronome |  |  |  | télévision |  |
|  | composer |  |  |  | présentateur |  |
|  | solo |  |  |  | télécommande |  |
|  |  |  |  |  |  |  |
| S2 | note |  |  | S2 | série |  |
|  | clé |  |  |  | diffuser |  |
|  | portée |  |  |  | film |  |
|  | partition |  |  |  | publicité |  |
|  | mélomane |  |  |  | journal |  |
|  |  |  |  |  |  |  |
| S1 | cueillir | **olere** |  | S1 | imperméable | **volore** |
|  | champignon |  |  |  | manteau |  |
|  | morille |  |  |  | parka |  |
|  | marché |  |  |  | veste |  |
|  | girolle |  |  |  | couvrir |  |
|  |  |  |  |  |  |  |
| S2 | cèpe |  |  | S2 | réchauffer |  |
|  | chanterelle |  |  |  | pardessus |  |
|  | ramasser |  |  |  | protéger |  |
|  | moisissure |  |  |  | poncho |  |
|  | truffe |  |  |  | blouson |  |

|  | **PRIME** | **TARGET** |  |  | **PRIME** | **TARGET** |
| --- | --- | --- | --- | --- | --- | --- |
|  |  |  |  |  |  |  |
| S1 | match | **noyon** |  | S1 | chocolat | **ideau** |
|  | adversaire |  |  |  | gourmandise |  |
|  | gagner |  |  |  | sorbet |  |
|  | tournoi |  |  |  | glace |  |
|  | vainqueur |  |  |  | douceur |  |
|  |  |  |  |  |  |  |
| S2 | raquette |  |  | S2 | déguster |  |
|  | arbitre |  |  |  | pâtisserie |  |
|  | filet |  |  |  | gâteau |  |
|  | tennis |  |  |  | biscuit |  |
|  | balle |  |  |  | meringue |  |
|  |  |  |  |  |  |  |
| S1 | géographie | **poma** |  | S1 | verser | **empro** |
|  | carte |  |  |  | contenir |  |
|  | européen |  |  |  | vider |  |
|  | climat |  |  |  | jatte |  |
|  | pays |  |  |  | carafe |  |
|  |  |  |  |  |  |  |
| S2 | continent |  |  | S2 | cruche |  |
|  | topographie |  |  |  | récipient |  |
|  | rivière |  |  |  | bocal |  |
|  | voyager |  |  |  | couler |  |
|  | montagne |  |  |  | amphore |  |
|  |  |  |  |  |  |  |
| S1 | miauler | **oppors** |  | S1 | éléphant | **ilone** |
|  | chat |  |  |  | safari |  |
|  | jouet |  |  |  | baobab |  |
|  | siamois |  |  |  | hyène |  |
|  | griffer |  |  |  | buffle |  |
|  |  |  |  |  |  |  |
| S2 | ronronnement | |  | S2 | zèbre |  |
|  | litière |  |  |  | jeep |  |
|  | souris |  |  |  | antilope |  |
|  | félin |  |  |  | savane |  |
|  | panier |  |  |  | braconner |  |

|  | **PRIME** | **TARGET** |  |  | **PRIME** | **TARGET** |
| --- | --- | --- | --- | --- | --- | --- |
|  |  |  |  |  |  |  |
| S1 | enfanter | **oumerd** |  | S1 | sorcière | **tola** |
|  | agneau |  |  |  | minotaure |  |
|  | gestation |  |  |  | raconter |  |
|  | chevreau |  |  |  | elfe |  |
|  | allaiter |  |  |  | fabuleux |  |
|  |  |  |  |  |  |  |
| S2 | veau |  |  | S2 | licorne |  |
|  | petit |  |  |  | dragon |  |
|  | poulain |  |  |  | légendaire |  |
|  | chaton |  |  |  | yéti |  |
|  | mamelle |  |  |  | comte |  |
|  |  |  |  |  |  |  |
| S1 | bricoler | **unerte** |  | S1 | ressentir | **oncere** |
|  | clou |  |  |  | sensible |  |
|  | tournevis |  |  |  | émotion |  |
|  | outils |  |  |  | angoisse |  |
|  | enduire |  |  |  | peur |  |
|  |  |  |  |  |  |  |
| S2 | ciment |  |  | S2 | colère |  |
|  | percer |  |  |  | éprouver |  |
|  | niveau |  |  |  | stress |  |
|  | scie |  |  |  | énerver |  |
|  | coller |  |  |  | tristesse |  |
|  |  |  |  |  |  |  |
| S1 | sandwich | **onas** |  | S1 | fusil | **opreck** |
|  | repas |  |  |  | cerf |  |
|  | boire |  |  |  | tirer |  |
|  | manger |  |  |  | abattre |  |
|  | serveur |  |  |  | biche |  |
|  |  |  |  |  |  |  |
| S2 | restaurant |  |  | S2 | chasser |  |
|  | bar |  |  |  | renard |  |
|  | déjeuner |  |  |  | sanglier |  |
|  | cuisinier |  |  |  | chevreuil |  |
|  | bistrot |  |  |  | belette |  |

|  | **PRIME** | **TARGET** |  |  | **PRIME** | **TARGET** |
| --- | --- | --- | --- | --- | --- | --- |
|  |  |  |  |  |  |  |
| S1 | ouvrage | **empru** |  | S1 | carte | **ospian** |
|  | carnet |  |  |  | tapis |  |
|  | écrire |  |  |  | distribuer |  |
|  | cahier |  |  |  | donne |  |
|  | page |  |  |  | mise |  |
|  |  |  |  |  |  |  |
| S2 | tome |  |  | S2 | jeton |  |
|  | chapitre |  |  |  | règle |  |
|  | essai |  |  |  | belotte |  |
|  | fiction |  |  |  | tricher |  |
|  | histoire |  |  |  | tarot |  |
|  |  |  |  |  |  |  |
| S1 | journal | **icerne** |  | S1 | sofa | **onquse** |
|  | chronique |  |  |  | chaise |  |
|  | reportage |  |  |  | allonger |  |
|  | lecteur |  |  |  | pouf |  |
|  | annoncer |  |  |  | méridienne |  |
|  |  |  |  |  |  |  |
| S2 | actualité |  |  | S2 | rembourrer |  |
|  | édito |  |  |  | coussin |  |
|  | courrier |  |  |  | siège |  |
|  | une |  |  |  | banc |  |
|  | impression |  |  |  | tabouret |  |
|  |  |  |  |  |  |  |
| S1 | laurier | **oprene** |  | S1 | pleuvoir | **umare** |
|  | thym |  |  |  | nuage |  |
|  | basilic |  |  |  | soleil |  |
|  | origan |  |  |  | météo |  |
|  | marjolaine |  |  |  | climat |  |
|  |  |  |  |  |  |  |
| S2 | ciboulette |  |  | S2 | température |  |
|  | sauge |  |  |  | neiger |  |
|  | fenouil |  |  |  | orage |  |
|  | parfumer |  |  |  | grêlon |  |
|  | assaisonner |  |  |  | averse |  |
